# Environmental Factors Influencing Species Richness Expression in Grasslands of the Colombian Orinoquia

**DOI:** 10.3390/plants13243545

**Published:** 2024-12-19

**Authors:** Vladimir Minorta-Cely, Larry Niño, Orlando Rangel, Daniel Sánchez-Mata

**Affiliations:** 1 Biology Program and Natural Sciences Services, Central University, Bogotá D.C. 111711, Colombia; vminortac@ucentral.edu.co; 2 Natural Science Institute, National University of Colombia, Bogotá D.C. 111321, Colombia; lninoa@unal.edu.co (L.N.); jorangelc@unal.edu.co (O.R.); 3 Botany Unit, Faculty of Pharmacy, Complutense University of Madrid, 28040 Madrid, Spain; 4 Harvard University Herbaria, Harvard University, Cambridge, MA 02138, USA

**Keywords:** neotropical savannas, flora and vegetation of the Colombian Orinoquia, cumulative probability ordinal and binary regressions, predictive richness models

## Abstract

The relationships between environmental characteristics and species richness in the grasslands of the Colombian Orinoquia are presented and analyzed using an ordinal logistic regression model. Ordinal and scale covariates were included, and their bivariate significance was assessed using Spearman’s rho and Kendall’s Tau-b. The covariates that showed statistical significance with the weighted richness thresholds (WRT) and defined the model were the soil depth and the soil moisture regime, both of which had positive correlations. In contrast, the percentage of bare soil and the monthly minimum temperature showed negative correlations. This contribution highlights the relevance of articulating and combining the floristic and phytosociological characterization of grassland vegetation to advance the predictive studies aimed at defining and understanding the potential divergent relationships between the vegetation and the basic attributes of the natural environment, along with their implications for conservation.

## 1. Introduction

The Neotropical savannas cover an area of approximately 2.5 million hectares. South of the Amazon, they include regions such as Beni (Bolivia), Cerrado (Brazil), and the Chaco (Paraguay); to the north lies the Orinoquia region, shared by Colombia and Venezuela [1,2,3,4,5]. Their ecological attributes are far from being extensively homogeneous, as there are clear geological, geomorphological, hydrological, climatic, topographic, and edaphic singularities that contribute to a biotic complexity of high conservation value [6,7,8,9,10,11,12,13,14].

Among the key contributions on the influence of habitat-defining factors on the richness and diversity of their plant communities is that of Bourlière and Hadley [15], who established relationships between vegetation structure and soil moisture, concluding that soil nutrients are a subordinate factor to climatic seasonality. Additionally, Sarmiento [16] documented the relationships between climatic seasonality, topography, and moisture regimes.

In the Brazilian cerrado, Goodland [17], Goodland and Pollard [18] and Lopes and Cox [19] documented the relationships between soil fertility gradients, leaf consistency, and the floristic composition of plant communities. They concluded that soil fertility is a decisive factor in explaining variations in species richness and floristic composition in these savannas. Pereira et al. [20] assessed the relationships between soil properties, species density, and species richness in savanna formations, concluding that soil characteristics (fertility, texture, and moisture) determine the geographic extent of vegetation formations, species abundance, and physiognomy. Menegat et al. [21] concluded that local edaphic and climatic variations, even within geographically proximate areas, significantly influence species richness and turnover in continuous vegetation formations. In the Beni savannas of Bolivia, Haase [22,23] concluded that precipitation regimes, soil moisture, and texture are the primary determinants of variations in species richness, structure, and floristic composition. Medina and Motta [24] identified the hydric gradient as the main factor explaining species richness and floristic composition. Van Donselaar [2] considered soil texture and moisture to be the most significant determinants of species richness and floristic composition in the Surinamese savannas. In the Venezuelan savannas, according to Sarmiento and Monasterio [25], geomorphology, parent material, soil depth, and drainage conditions showed the highest correlation with vegetation richness and diversity. Frost et al. [26] concluded that soil moisture and nutrient availability are the key factors in explaining species richness and floristic composition, with human activities identified as external modifying agents. Sarmiento and Pinillos [27] and Sarmiento et al. [28] established the relationship between geomorphology and hydrology with species richness and floristic composition. According to Chacón-Moreno et al. [29], soil fertility is not a determining factor, but water dynamics and water retention capacity are the principal determinants of species richness and distribution. Baruch [9] attributed the highest correlation with species richness to moisture and fertility indices, establishing that precipitation amounts and distribution, nutrient availability, and edaphic factors are the environmental determinants most influential in species richness, physiognomy, and levels of association in seasonal savannas.

Regarding the species richness and floristic composition of the Colombian Orinoquian grasslands, data indicate the presence of approximately 4800 species (Rangel-Ch. et al., in prep.). The phytosociological characterization encompasses one (1) class, three (3) orders, nineteen (19) alliances, and sixty-three (63) associations. In the high plains, the associations are composed, on average, of twenty-eight (28) characteristic/differential species, with notable contrasts in richness values, such as *Desmodio barbati*-*Sipanetum pratensis* (49 species) and *Panico pilosi-Schizachyrietum brevifoli* (13 spp.). In the alluvial plains, associations consist of an average of 38 species, with *Ludwigio erectae-Axonopodetum purpusi* (64 spp.) and *Axonopodo ancepitis*-*Curatelletum americanae* (22 spp.) standing out [5,30,31,32].

In contrast, the relationships between species richness expression in these vegetation types and the basic attributes of the natural environment have been scarcely addressed at the local scale and even less at the regional level. Beard [33] highlighted the influence of nutrient availability on species richness and structure. Other studies [1,32,34,35,36,37,38,39,40,41,42,43,44,45,46,47,48] provide a partial overview of the influence of edaphic conditions, flooding, and waterlogging on richness and diversity values.

Despite prior studies, there is no consensus regarding which set of ecological factors holds the greatest relevance for the floristic richness of the Colombian Orinoquian grasslands. The role of climatic and edaphic factors in shaping floristic richness and composition remains unclear. It is proposed that species richness in the grasslands of the Orinoco region is significantly influenced by climatic and edaphic factors. Specifically, temperature, precipitation, and soil characteristics such as texture, moisture, and aluminum content interact to shape species richness. Variations in these factors are expected to explain patterns of distribution and richness, demonstrating that certain combinations of climatic and edaphic conditions promote greater biodiversity expression in grasslands.

Therefore, it is necessary to conduct a more in-depth analysis of these factors to understand their impact on the floristic significance of the region. Thus, the primary objective of this manuscript is to propose a model for the phytocoenoses described in the Colombian Llanos, with the goal of addressing: (i) which set of ecological factors is most relevant to their floristic richness? and (ii) how do these attributes interact, and what is their influence on species richness values?

To address these questions, this study proposes the following hypothesis based on the 292 vegetation plots in the Colombian Orinoquian grasslands: climate and soil factors are the primary environmental attributes, and play different roles in shaping their floristic composition. These models are essential for deepening the understanding of the region, as they allow for a detailed description of its environmental and biotic singularities. Furthermore, they provide key inputs for the development of thematic maps and delineation of strategic (ecogeographic or biogeographic) areas for biodiversity conservation, such as national parks and newly protected areas, among others.

## 2. Results

Table 1 shows the frequencies of the dependent variable, corresponding to the weighted richness category, those considered as factors (nominal variables), and covariates (ordinal variables with their respective ordinal numerical reclassification). Table 2 presents the descriptive statistics of the scale covariates.

According to the bivariate exploratory analysis, the statistically significant covariates exhibited an inverse correlation with the weighted richness, with the exception of soil depth. Based on the magnitude of the statistically significant correlations, the percentage of bare soil showed the highest correlation with weighted richness, followed by minimum monthly precipitation, minimum monthly temperature, soil depth, maximum monthly temperature, annual mean precipitation, and soil moisture regime, respectively (Table 3).

In the bivariate Wald statistics, all values, except for minimum monthly temperature, were greater than two, indicating statistical significance; these results align with the significant associations in Table 3. Regarding VIF analysis, annual mean temperature and maximum monthly precipitation were removed due to their strong collinearity with maximum monthly temperature and annual mean precipitation, respectively. These exclusions ensured that the retained variables provided unique and independent contributions to the model, minimizing redundancy. By addressing multicollinearity, this step also improved the precision of estimated coefficients and the overall reliability of the ordinal regression models, enabling a clearer understanding of how climatic and edaphic factors are related to species richness. In the multivariate model, three of the Wald values are not significant, implying that in both the bivariate and multivariate exploratory analyses, the covariates of maximum monthly temperature, minimum monthly temperature, and annual mean precipitation do not contribute to the regression. Therefore, these variables should be eliminated to achieve a more parsimonious model. According to the error values, the variables with significant Wald statistics show disparate coefficients in the multivariate model compared to the bivariate model. This suggests that the relationship of these variables with weighted richness is affected by an interaction or confounding effect, as the statistical results depend on the inclusion or exclusion of other variables from the model (Table 4).

The initial and final model fits, represented by the values of −2 log-likelihood (−2LL) and a Chi-square test on their difference (statistic = 38.795; significance = 0.000), indicating that the regression provides a significant improvement with the variables included in the final model (−2LL = 523.243) compared to the baseline or intercept model with only the constant (−2LL = 562.037).

The Cox and Snell R^2^ for the ordinal regression was calculated at 0.129, suggesting that 12.9% of the variation in weighted richness is attributed to the included covariates. The Nagelkerke R^2^ represents a scale adjustment of the Cox and Snell R^2^. The calculated value for the ordinal regression was 0.143, estimating that 14.3% of the variation in the dependent variable can be explained by the predictor variables. Although the pseudo R^2^ values were modest, these statistics are indicative between similar models but not conclusive, as none of them explain variance analogously to the R^2^ coefficient of linear regressions. The multicollinearity test indicated that the assumption of equal coefficients for all response categories is not rejected, thereby validating the feasibility of the ordinal procedure.

Table 5 shows the parameter estimates that summarize the effects of each predictor included in the grassland analysis. The statistical significance (<0.05) of the covariates related to soil depth, percentage of bare soil, and minimum monthly temperature suggest that the effect observed on weighted richness is not attributable to chance. Conversely, the significance of the soil depth covariate contributes little to the model, although it is close to the established cutoff point; this could be considered a marginally significant variable and may warrant retention in the regression, as the effects of covariates are cumulative and provide useful information.

According to the regression slope coefficients, both the soil depth and the soil moisture regime exhibit positive values, indicating that as their magnitudes increase, the probability of being classified in a higher category of weighted richness also increases; in other words, greater depth and moisture levels are associated with a higher number of species. Conversely, the percentage of bare soil and the minimum monthly temperature show negative coefficients, suggesting that an increase in their magnitudes corresponds to a higher probability of being classified in a lower category of weighted richness; thus, a greater proportion of bare soil and a higher temperature during the hottest month are associated with a lower number of species.

The model estimates the cumulative probabilities for each category of weighted richness, which are utilized in selecting the most probable outcome for each case. The calculation of these probabilities is performed using the predictor values in the model equations and the inverse of the link function, defined as the negative log-log based on the definitions of the variables. The probabilities for the individual categories of weighted richness can be estimated by sequentially taking the differences of the cumulative probabilities; specifically, the probability for the first category corresponds to the first cumulative probability, the probability for the second category is derived by subtracting the first cumulative probability from the second, and so on.

In line with the above, and utilizing the parameters from the ordinal regression, it is feasible to establish the model equations, which facilitate the calculation of the probability of belonging to each of the four categories of weighted richness, based on the link function (negative log-log) and the three thresholds (Table 5), which are defined below.

### Model

- WRT = 1:

−log(−log(WRT1)) = 0.333 − (SOIL_DEPTH*0.311) − (SOIL_MOIST*0.154) − (BARE_SOIL*−0.027) − (MIN_MONTH_TEMP* − 0.067).(1)

- WRT = 2:

−log(−log(WRT2)) = 1.599 − (SOIL_DEPTH*0.311) − (SOIL_MOIST*0.154) − (BARE_SOIL*−0.027) − (MIN_MONTH_TEMP* − 0.067).(2)

- WRT = 3:

−log(−log(WRT3)) = 4.297 − (SOIL_DEPTH*0.311) − (SOIL_MOIST*0.154) − (BARE_SOIL*−0.027) − (MIN_MONTH_TEMP* − 0.067).(3)

## 3. Discussion

### 3.1. Importance of Predictive Richness Models

The floristic composition and species richness of grassland communities are governed by many ecological drivers such as climate, soil moisture, humidity, and nutrient availability [49]. The relationships among these processes, environmental factors, and biocenoses have been extensively examined through various ecological models, emphasizing that the variation within phytocenoses is a direct response to local singularities and subsequently to regional heterogeneity (see, among others, [49,50,51,52,53,54,55]). Predictive richness models applied in ecology and vegetation science primarily focus on resolving how the fundamental attributes of the natural environment are differentially and asymmetrically combined in ostensibly homogeneous areas. Additionally, these models aim at clarifying the set of processes that define the richness and diversity of plant communities. In the case of grasslands (primarily Asian and European), their applications are multiscale and widely utilized in studies concerning species distribution patterns, population and community ecology, ecological niche characterization, flora and vegetation mapping, carbon capture, functional ecology, among others [49,50,55,56,57,58,59,60,61,62,63].

The model proposed herein estimates the probability and addresses questions regarding the primary factors affecting the expression of floristic richness in Colombian Orinoco grasslands. This opens up possibilities for advancing predictive studies aimed at defining and understanding potential divergent relationships (positive, negative, or incipient) between vegetation (zonal, azonal, or extrazonal) and the fundamental attributes of the natural environment. According to the bivariate exploratory analysis, the covariables that exhibited statistical significance concerning the thresholds of weighted richness (WRT) were soil depth, soil moisture regime, percentage of bare soil, minimum monthly temperature, maximum monthly temperature, mean annual precipitation, and minimum monthly precipitation. In the statistical contrast of the bivariate and multivariate regressions, the covariables of minimum monthly temperature, maximum monthly temperature, and mean annual precipitation were excluded. In the ordinal regression, the covariables defining the richness model were soil depth and soil moisture regime, both of which showed positive correlations. Conversely, the percentage of bare soil and minimum monthly temperature demonstrated negative correlations.

Our analysis reveals that environmental characteristics, particularly soil conditions and climatic variables, significantly impact the species richness of the studied grasslands. Specifically, a higher percentage of bare soil and elevated minimum monthly temperature are associated with lower species richness, suggesting that these factors limit plant diversity. On the other hand, greater soil depth correlates positively with species richness, indicating that deeper soils provide more favorable conditions for the establishment and development of a larger number of species. Furthermore, this model allows for an understanding of how these factors interact in accordance with the ecological particularities that define the physiographic units of the region, and what their effects are on the richness values, floristic composition and ecogeographical extent of its phytocenoses.

### 3.2. Ecological Aspects

Climate serves as the primary ecological filter at the regional scale, while variations in soil composition represent a more specific filter operating at the local level. This binomial influences richness, floristic composition, vegetation structure, and the autoecology of its species. Seasonality, precipitation amounts, and their distribution impact edaphic conditions (texture, fertility, pH). Water runoff consolidates substrates of variable nature (deficiency or excess of water), forming physiological barriers [64,65]. These aspects have been discussed, among others [66,67,68,69], where precipitation over other environmental factors was emphasized as the principal element defining the physiognomy, structure, and expression of richness in savanna formations.

The model proposed herein aligns with the findings of Peng et al. [70] and Qian et al. [71], which underscore how monthly fluctuations in precipitation amounts, soil composition, and topographical characteristics are closely related to geographical variations in vegetation types and, subsequently, their richness. It also corresponds with the considerations of Toledo et al. [72], who detail how the richness of grasslands is affected in the following order: precipitation > temperature > soil fertility > soil texture. Additionally, it partially conforms to the contributions of Sarmiento [73,74], Hasse [22,23], Solbrig et al. [75], and Anadon et al. [76], who highlight the relationships among topography, soil texture, moisture, water retention capacity, nutrients, climate, and their impact on the expression and significance of richness in neotropical savannas.

When stochastic scenarios prevail, floristic composition, richness, and the structure of plant communities exhibit considerable variability [54,77,78]. Numerous studies assess the combined effects of climatic and hydric variations alongside anthropogenic disturbances, as well as their applications for delineating conservation areas [79,80]. Models serve as predictive alternatives regarding how biota differentially interacts according to the fundamental attributes of the natural environment; however, it is crucial to emphasize the importance of recognizing and delving into the highly complex temporal effects, stress agents, and intrinsic dynamics of natural systems, which significantly impact their interpretation [49,80,81,82].

## 4. Materials and Methods

### 4.1. Study Area

The Colombian Orinoquia spans areas within the departments of Arauca, Casanare, Meta, and Vichada, covering approximately 17 million hectares (ca. 30% of the national territory), with elevations ranging between 180 and 675 m above sea level, featuring a slight slope towards the Northeast [38,44,83,84]. The tectonic conditions that led to its formation are associated with the rise of the Eastern Cordillera during the mid-Tertiary (Oligocene-Miocene) in two main fronts: a longer one between the Duda and Upía rivers, and a shorter, yet equally intense one in the El Cocuy region, along with continuous sedimentation through a river system that descended from the new mountainous system [35,85].

This region originated from a large geosyncline of alluvial sedimentary origin, influenced by tectonic events, Quaternary climate, ancient erosive processes in the Guiana Shield, and recent erosion from the Andes [86,87,88,89,90]. Although topographic contrasts are minimal, a complex mosaic of soils and micro-reliefs prevails, resulting from differential processes in the origin, age of the parent material, sedimentation, and land dissection [83]. Despite being recognized as a relatively homogeneous territory, with gentle slopes and predominantly covered by grasses [91], its physiography is closely related to chronological criteria, the distance from the Eastern Cordillera, and the Meta River, which demarcates drainage conditions influencing the relief variations: the poorly drained Orinoquia to the north of the river, characterized by fans and alluvial and aeolian plains, and the well-drained Orinoquia to the south, featuring predominantly flat and undulating high plains, hills, and terraces [1,34,41,44]. The sedimentary materials, both from the Tertiary and Quaternary periods, were deposited on an ancient platform belonging to the Guiana Shield, composed of Precambrian rocks aged between 1 and 1.8 billion years, with outcrops prominently seen in La Macarena and the high plains, forming mountain ranges and isolated hills near the Guaviare and Orinoco rivers, known as the residual Altillanura [84,90,91,92].

The Orinoquia is characterized by climatic homogeneity, influenced by its flat topography and rainfall–temperature patterns. The rainfall distribution in the Colombian section follows a bi-seasonal unimodal regime with a brief transition. During the first and last quarters of the year, drought conditions prevail, while from April to October, maximum humidity levels are recorded, representing between 50.7% and 88.5% of the annual precipitation [93]. According to various climate classifications [94], the region is defined as follows: (i) according to Caldas-Lang, it has a warm–humid climate in the Piedmont and warm–semi-humid climate in the rest of the region; (ii) according to Martonne, La Macarena experiences a wet rainy climate without seasonal variations, while the Piedmont is wet and rainy, and the Altillanura and Inundable Plains are simply wet; (iii) Thornthwaite identifies a climatic gradient extending from the Piedmont towards Venezuela in a northeast direction, ranging from wet, moderately wet, slightly wet to semi-humid climates; (iv) Köeppen classifies the Piedmont as a tropical rainforest climate, the northeast as a tropical savanna climate, and the rest of the Orinoquia as a tropical rainforest climate. It is important to note that although these classifications are based on a systematic grouping of climatic elements and have proven effective in the contexts for which they were created, they have shown inconsistencies when applied to other regions [95].

**Figure 1:**
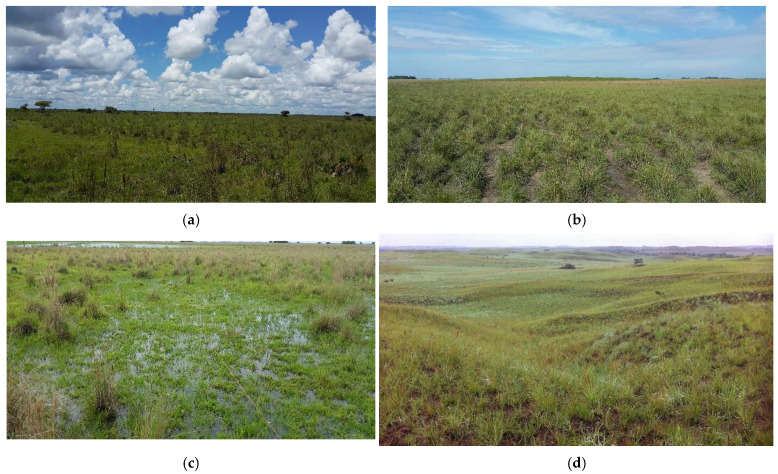

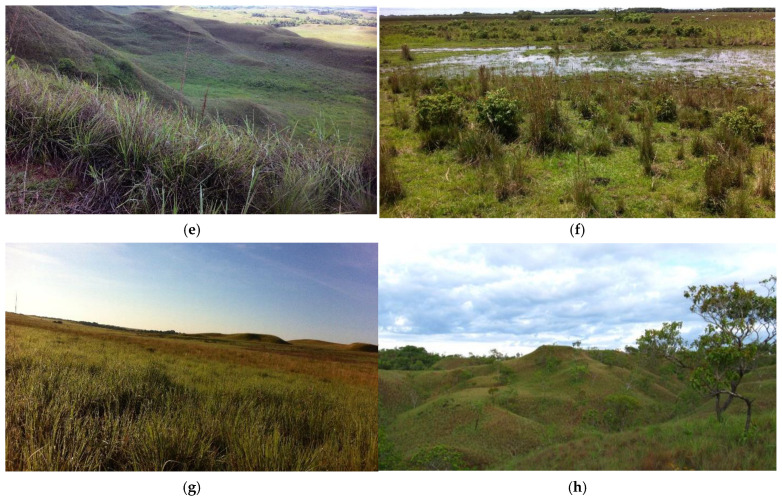
Physiography in grasslands and meadows of the alliances: (**a**) *Paspalion carinato—pectinati* in High plain; (**b**) *Steinchismo laxae—Andropogonion bicornis* in Alluvial plain; (**c**) *Rhynchosporo barbatae—Andropogonion virgarti* in Alluvial plain; (**d**) *Axonopodo aurei-Trachypogonion spicati* in High plain; (**e**) *Paspalo pectinati—Axonopodion aurei* in High plain; (**f**) *Andropogono virgati—Axonopodion ancepitis* in Alluvial plain; (**g**) *Hyptio brachiatae—Trachypogonion spicati* in High plain; (**h**) *Bowdichio virgilioidis—Curatellion americanae* in High plain.

In the Highplains, differential sedimentation processes, coupled with rainfall dynamics, have shaped environments with varying levels of dissection. Along the edges of the interfluves and on the less dissected terraces, extensive stretches of shrubs and grasslands emerge, connecting with forested areas. In well-drained zones with differing degrees of dissection, banks dominated by grassland–herbaceous formations extend to the bases of hills, where grasslands, herbaceous vegetation, and shrublands form transitional zones between waterlogged or flooded areas and the large dissected terraces, which are dominated by grasslands, savannas, and shrublands. In the alluvial plain, fluvial, fluviolacustrine, and alluvial processes have created environments significantly influenced by the seasonal dynamics of precipitation regimes. This has led to the presence of flood-prone grasslands (overflow “*bajos* or *bajíos*”) and waterlogged areas (phreatic “*bajos* or *bajíos”*), interspersed irregularly with seasonal, semi-seasonal, and permanent marshes and swamps, dominated by herbaceous vegetation and some low-growing shrubs [30,31,32] (Figure 1).

### 4.2. Dataset

For the construction of the model, 292 georeferenced grassland surveys were considered ([32,96]; Table A1). The categories of weighted richness were established according to [97] and based on the natural break classification of the number of species recorded in each survey, where the data were grouped according to their inherent characteristics to define intervals of similar values and maximize the differences between classes [98]. This approach, in addition to providing a more uniform structure to a set of surveys derived from various studies (floristic, geobotanical, phytosociological, ecological, etc.), allows for a preliminary understanding of how richness values vary and segregate hierarchically within syntaxonomic arrangements, without the variability in the number of surveys per physiographic unit significantly interfering with their interpretation at the regional scale.

Reclassifying numerical species richness into ordinal categories such as paucispecific, oligospecific, mesospecific, and polyspecific offers several advantages, particularly when working with small datasets and exploring ecological hypotheses. It simplifies the analysis by reducing noise and making models statistically more robust, especially for ordinal regression methods that are better suited for limited samples. Additionally, it enhances ecological interpretation by capturing qualitative shifts in biodiversity and linking them to specific climatic and edaphic thresholds. This approach also mitigates issues related to sparse data in certain richness ranges, enabling the detection of broader patterns. Finally, it facilitates communication of results, as the ordinal categories are more intuitive and actionable for conservation and management purposes.

Species richness was reclassified into ordinal categories using geometric intervals. It is justified from both statistical and ecological perspectives, especially when working with small datasets. Geometric intervals divide data proportionally on a scale that reflects the nonlinear patterns typical of ecological variables. This approach emphasizes relative differences at lower richness levels, where biodiversity changes have greater ecological significance, while smoothing variability at higher levels. It ensures a balanced distribution of categories, preventing overrepresentation of certain ranges, which is particularly important for small datasets. Additionally, geometric intervals provide a consistent and transparent method for classification, enhancing the interpretability and comparability of results.

The percentage of bare soil data was collected directly in the field, slope data were extracted from the processing of the Shuttle Radar Topography Mission (SRTM v.3) 30 m digital elevation model, climate data for temperature and precipitation were extracted from WorldClim bioclimatic models [99], and other data related to physiography, relief, and edaphic characteristics were obtained from Colombia’s geopedology map at a 1:100,000 scale [100].

### 4.3. Statistical Framework

As the response variable, plant species richness in the Orinoquia grasslands was used, classified into four alpha diversity categories: paucispecific, oligospecific, mesospecific, and polyspecific. Based on the structure of the ordinal dependent variable, the negative log−log (−log(−log(x))) link function was established, as it is typically applied when lower categories are more likely in the variable’s distribution [101]. The analysis included both ordinal and scale covariates. The ordinal covariates considered were soil depth, with categories ranging from shallow to deep; soil texture, from fine to coarse; soil moisture regime, from ustic to aquic; and three levels of aluminum content. The scale covariates included the percentage of bare soil, annual mean temperature, monthly maximum temperature, monthly minimum temperature, annual mean precipitation, monthly maximum precipitation, monthly minimum precipitation, and slope percentage [32].

A multivariate approach was employed for data analysis. Initially, a bivariate exploratory analysis was conducted using Spearman’s Rho to assess the correlation between scale covariates and weighted richness. For ordinal covariates, Kendall’s Tau-b was applied. Only those covariates with a two-tailed bivariate significance level of less than 0.05 were selected for multivariate analysis. Additional exploratory analyses were then performed through bivariate and multivariate ordinal logistic regressions. Before conducting the exploratory ordinal regressions, a Variance Inflation Factor (VIF) test was performed to evaluate multicollinearity among the predictor variables. High multicollinearity can inflate standard errors, reduce the statistical significance of predictors, and complicate the interpretation of regression coefficients, potentially leading to unreliable models. Variables with VIF values exceeding 10, a commonly used threshold indicating severe multicollinearity, were excluded to improve model stability and robustness.

In the multivariate regressions, variables showing statistical significance in the bivariate regressions were included. The final variable selection was based on Wald statistics. Covariates that did not contribute significantly to the regression were excluded from the final model. The final ordinal regression model included the selected covariates and was evaluated using −2 log likelihood (−2LL) and the Chi-square test, comparing the initial and final model fits. Cox and Snell’s R^2^ and Nagelkerke’s R^2^ values indicated the model’s validity in predicting variation in weighted richness. A two-tailed bivariate significance of <0.05 with weighted richness was used as the inclusion criterion for an independent variable in the multivariate analysis. The correlation of scale covariates was estimated with Spearman’s rho, while the correlation of ordinal covariates was assessed using Kendall’s Tau-b. Factors, as nominal variables, were not considered in the bivariate exploratory analysis. To compare the behavior of regression coefficients, complementary exploratory analyses were conducted with bivariate and multivariate ordinal logistic regressions, where variables meeting the established statistical significance during the bivariate analysis were included. Once the variables to be included in the final model were determined based on the statistical significance estimated during the bivariate and multivariate exploratory analyses, an ordinal regression was conducted [32,102].

## 5. Conclusions

Only through a comprehensive and multidimensional perspective of biodiversity it is possible to design and implement effective conservation actions that are appropriate to the sociocultural context of the territories. Over the past decade, there has been a notable global interest in deepening the understanding of grassland richness, highlighting the ecological and sociocultural implications that must be considered for its conservation [103,104,105,106]; consequently, accurate characterization (both biophysical and cultural) serves as the primary starting point for the development of plans and actions aimed at these purposes.

Multiple physical processes operate at different scales in nature, creating gradients that limit the extent and meanings of the ecological expression of species and, subsequently, of plant communities. It is possible that these gradients largely exceed the statistical models that aim to explain the vegetation–environment relationships [107]. While phytocenoses are a direct response to the characteristics defining the habitat assemblages present in the Colombian Llanos, their ecogeographical reach and biotic uniqueness cannot be inferred under the premise of regional homogeneity.

Our findings support the hypothesis that environmental factors and soil physical properties play a significant role in the patterns of plant species richness in grasslands. In this regard, similar relationships have been described in various regions of Africa, with particular emphasis on how nitrogen levels, along with soil texture and, subsequently, moisture, influence species diversity [108]. In our study area, soil depth, soil moisture as a result of its structural attributes (texture), and the percentage of bare soil are the key factors that help outline and explain a regional pattern regarding the prevalence of grasslands tending toward oligospecific to mesospecific conditions. This pattern is comparable to other grassland-dominated areas, such as the *páramos*, and to a lesser extent, the casmophytic savannas of the Guayana region.

This contribution underscores the relevance of articulating and combining the floristic and phytosociological characterization of grassland vegetation with the fundamental attributes of the natural environment for constructing predictive models of species richness. Additionally, it emphasizes the importance of conducting regional and local studies on the flora, vegetation, and ecology of the Colombian eastern extrandine grasslands, which possess a biogeographical and paleoecological significance that remains unresolved. Although the use of such models is primarily associated with specific studies aimed at addressing issues related to Alpha diversity and, to a lesser extent, Beta diversity, they can also be extended to strengthen ecological niche models, accurately assess the biotic significance of territories, analyze the spatial and temporal dynamics of plant communities, and, of course, identify new areas designated for conservation. In this regard, the data and information used in this contribution were pivotal in supporting the process of declaring a new national natural park, the Serranía de Manacacías National Natural Park (Meta department), and are currently being used in the process of establishing new conservation areas such as the Arauca Wetlands (Arauca department) and the Transitional Forests of Cumaribo (Vichada department).

## Figures and Tables

**Table 1:** Frequencies of nominal and ordinal variables considered in the Colombian Orinoquian grassland’s floristic richness analysis.

	Categories (Ordinal Reclassification)	Frequency	Percentage
Alpha Diversity Category (Richness: dependent variable)	Paucispecific, 0–9 species (1)	104	37.0
	Oligospecific, 10–14 species (2)	103	36.7
	Mesospecific, 15–21 species (3)	68	24.2
	Polyspecific, 22–38 species (4)	6	2.1
Soil Depth (covariate)	Shallow (1)	55	19.6
	Shallow to Moderately Shallow (2)	22	7.8
	Moderately Shallow to Moderately Deep (3)	109	38.8
	Moderately Deep (4)	62	22.1
	Moderately Deep to Deep (5)	1	0.4
	Deep (6)	32	11.4
Soil Texture (covariate)	Fine (1)	161	57.3
	Fine to Medium (2)	4	1.4
	Fine to Coarse (3)	17	6
	Medium and Fine (4)	43	15.3
	Medium (5)	1	0.4
	Coarse and Fine (6)	18	6.4
	Coarse and Medium (7)	5	1.8
	Coarse (8)	32	11.4
Soil Moisture Regime (covariate)	Ustic (1)	6	2.1
	Ustic and Udic (2)	2	0.7
	Udic and Ustic (3)	30	10.7
	Udic (4)	61	21.7
	Udic and Aquic (5)	111	39.5
	Aquic and Udic (6)	22	7.8
	Aquic (7)	49	17.4
Aluminum Level (covariate)	High (1)	213	75.8
	Medium (2)	17	6
	Low (3)	51	18.1

**Table 2:** Descriptive statistics of the ecological scalar variables in the Colombian Orinoquian grassland’s floristic richness analysis.

Scalar Covariate	Range	Minimum	Maximum	Mean	Standard Error	Standar Deviation	Variance
Percentage of Bare Soil	77	0	77	12.04	0.881	14.766	218.049
Mean Annual Temperature (°C)	3.6	24.7	28.3	27.106	0.0177	0.2966	0.088
Maximum Monthly Temperature (°C)	4.6	31.7	36.3	34.582	0.029	0.4861	0.236
Minimum Monthly Temperature (°C)	3.7	19.1	22.8	21.727	0.0198	0.3315	0.11
Mean Annual Precipitation (mm)	1453	1605	3058	2253.19	11.545	193.529	37,453.289
Maximum Monthly Precipitation (mm)	180	258	438	337.5	1.284	21.516	462.958
Minimum Monthly Precipitation (mm)	25	6	31	15.66	0.357	5.984	35.811
Percentage of Terrain Slope	17.4	0	17.4	1.974	0.1554	2.6046	6.784

**Table 3:** Statistics from the ecological bivariate exploratory analysis in the Colombian Orinoquian grassland’s floristic richness.

Covariate	Tau-b	Rho	Sig. (Bilateral)
Soil Depth	0.140		0.006 *
Soil Texture	0.027		0.609
Soil Moisture Regime	−0.107		0.035 *
Aluminum Level	0.071		0.198
Percentage of Bare Soil		−0.247	0.000 *
Mean Annual Temperature		−0.077	0.201
Maximum Monthly Temperature		−0.129	0.031 *
Minimum Monthly Temperature		−0.159	0.008 *
Mean Annual Precipitation		−0.127	0.034 *
Maximum Monthly Precipitation		0.024	0.686
Minimum Monthly Precipitation		−0.159	0.008 *
Percentage of Terrain Slope		−0.055	0.362

* Significance (Sig.) at 0.05 level.

**Table 4:** Comparison of bivariate and multivariate exploratory regression in the Colombian Orinoquian grassland’s floristic richness analysis.

	Ordinal Regression Statistics (Bivariate/Multivariate)		
Covariate	Coefficient	Error	Wald
Soil Depth	0.149/0.303	0.052/0.082	9.345/13.536
Soil Moisture Regime	−0.105/0.156	0.056/0.091	3.444/2.955
Percentage of Bare Soil	−0.017/−0.028	0.006/0.007	7.743/17.180
Maximum Monthly Temperature	−0.239/−0.359	0.154/0.342	2.407/1.101
Minimum Monthly Temperature	−0.309/0.431	0.224/0.542	1.902/0.633
Mean Annual Precipitation	−0.001/0.001	0.000/0.001	2.770/1.453
Minimum Monthly Precipitation	−0.031/−0.100	0.013/0.033	5.776/9.135

**Table 5:** Statistics from the ordinal regression for weighted richness in the Colombian Orinoquian grassland’s floristic richness.

		Coefficient	Error	Wald	gl	Sig.	Confidence Interval 95%	
							Lower Bound	Upper Bound
Weighted Richness Thresholds (WRT)	WRT = 1	0.333	0.645	0.267	1	0.605	−0.931	1.597
	WRT = 2	1.599	0.654	5.986	1	0.014	0.318	2.881
	WRT = 3	4.297	0.763	31.723	1	0.000	2.802	5.793
Covariates	Soil Depth (SOIL_DEPTH)	0.311	0.081	14.807	1	0.000	0.152	0.469
	Soil Moisture Regime (SOIL_MOIST)	0.154	0.089	3.007	1	0.083	−0.02	0.329
	Percentage of Bare Soil (BARE_SOIL)	−0.027	0.006	17.871	1	0.000	−0.04	−0.015
	Minimum Monthly Temperature (MIN_MONTH_TEMP)	−0.067	0.015	21.257	1	0.000	−0.095	−0.039

## Data Availability

All data supporting the reported results are included in the paper.

## Appendix A

**Table A1:** Geographic location of the Colombian Orinoquian grassland plots. Area 10 × 10 m.

Id_Plot	Plot	Physiography	Location	Spp. No.	pH	Parent Material	Precipitation mm	Slope	Vegetation Cover (%)	Bare Soil (%)	Longitude	Latitude	Altitude
1	S.11	High plain	Manacacías	9	4.0–5.5	Clay alluvium	>2300	>2	98	2	72°27′16.95″	3°29′52.3″	172
2	S.13	High plain	Manacacías	10	4.0–5.5	Clay alluvium	>2300	>2	99	1	72°27′16.97″	3°29′52.3″	172
3	S.15	High plain	Manacacías	14	4.0–5.5	Clay alluvium	>2300	>2	99	1	72°27′16.99″	3°29′52.3″	172
4	S.17	High plain	Manacacías	10	4.0–5.5	Clay alluvium	>2300	>2	94	6	72°27′16.1″	3°29′52.3″	172
5	S.18	High plain	Manacacías	15	4.0–5.5	Clay alluvium	>2300	>2	98	2	72°27′16.1″	3°29′52.3″	172
6	S.14	High plain	Manacacías	11	4.0–5.5	Clay alluvium	>2300	>2	100	0	72°27′16.98″	3°29′52.3″	172
7	S.12	High plain	Manacacías	17	4.0–5.5	Clay alluvium	>2300	>2	99	1	72°27′16.96″	3°29′52.3″	172
8	S.39	High plain	Manacacías	5	4.0–5.5	Clay alluvium	>2300	>2	96	4	72°24′16.12″	3°26′45.02″	169
9	S.21	High plain	Manacacías	11	4.0–5.5	Clay alluvium	>2300	>2	96	4	72°24′16.11″	3°26′46.32″	166
10	S.22	High plain	Manacacías	8	4.0–5.5	Clay alluvium	>2300	>2	100	0	72°24′16.11″	3°26′46.32″	166
11	S.23	High plain	Manacacías	10	4.0–5.5	Clay alluvium	>2300	>2	99	1	72°24′16.11″	3°26′46.32″	166
12	S.24	High plain	Manacacías	7	4.0–5.5	Clay alluvium	>2300	>2	100	0	72°24′16.11″	3°26′46.32″	166
13	S.25	High plain	Manacacías	7	4.0–5.5	Clay alluvium	>2300	>2	100	0	72°24′16.11″	3°26′46.32″	166
14	S.26	High plain	Manacacías	7	4.0–5.5	Clay alluvium	>2300	>2	96	4	72°24′16.11″	3°26′46.32″	162
15	S.1	High plain	Manacacías	8	4.0–5.5	Clay alluvium	>2300	>2	95	5	72°27′16.85″	3°29′50.53″	169
16	S.2	High plain	Manacacías	7	4.0–5.5	Clay alluvium	>2300	>2	92	8	72°27′16.86″	3°29′50.53″	169
17	S.10	High plain	Manacacías	7	4.0–5.5	Clay alluvium	>2300	>2	96	4	72°27′16.94″	3°29′50.53″	170
18	S.5	High plain	Manacacías	7	4.0–5.5	Clay alluvium	>2300	>2	99	1	72°27′16.89″	3°29′50.53″	169
19	S.6	High plain	Manacacías	4	4.0–5.5	Clay alluvium	>2300	>2	92	8	72°27′16.9″	3°29′50.53″	170
20	S.7	High plain	Manacacías	5	4.0–5.5	Clay alluvium	>2300	>2	100	0	72°27′16.91″	3°29′50.53″	170
21	S.9	High plain	Manacacías	5	4.0–5.5	Clay alluvium	>2300	>2	99	1	72°27′16.93″	3°29′50.53″	170
22	S.19	High plain	Manacacías	4	4.0–5.5	Clay alluvium	>2300	>2	28	72	72°27′16.1″	3°29′52.3″	172
23	S.27	High plain	Manacacías	8	4.0–5.5	Clay alluvium	>2300	>2	100	0	72°24′16.11″	3°26′46.32″	162
24	S.30	High plain	Manacacías	7	4.0–5.5	Clay alluvium	>2300	>2	95	5	72°24′16.11″	3°26′46.32″	162
25	S.3	High plain	Manacacías	6	4.0–5.5	Clay alluvium	>2300	>2	95	5	72°27′16.87″	3°29′50.53″	169
26	S.8	High plain	Manacacías	4	4.0–5.5	Clay alluvium	>2300	>2	100	0	72°27′16.92″	3°29′50.53″	170
27	S.61	High plain	Manacacías	8	4.0–5.5	Clay alluvium	>2300	>2	99	1	72°26′16.15″	3°30′35.75″	183
28	S.64	High plain	Manacacías	8	4.0–5.5	Clay alluvium	>2300	>2	99	1	72°26′16.15″	3°30′35.75″	183
29	S.65	High plain	Manacacías	7	4.0–5.5	Clay alluvium	>2300	>2	100	0	72°26′16.15″	3°30′35.75″	183
30	S.66	High plain	Manacacías	7	4.0–5.5	Clay alluvium	>2300	>2	99	1	72°26′16.15″	3°30′35.75″	180
31	S.67	High plain	Manacacías	9	4.0–5.5	Clay alluvium	>2300	>2	88	12	72°26′16.15″	3°30′35.75″	180
32	S.71	High plain	Manacacías	16	4.0–5.5	Clay alluvium	>2300	>2	100	0	72°26′16.16″	3°30′29.27″	200
33	S.73	High plain	Manacacías	19	4.0–5.5	Clay alluvium	>2300	>2	100	0	72°26′16.16″	3°30′29.27″	200
34	S.75	High plain	Manacacías	12	4.0–5.5	Clay alluvium	>2300	>2	100	0	72°26′16.16″	3°30′29.27″	200
35	S.101	High plain	Manacacías	16	4.0–5.5	Clay alluvium	>2300	>2	99	1	72°26′16.19″	3°30′29.27″	186
36	S.102	High plain	Manacacías	21	4.0–5.5	Clay alluvium	>2300	>2	98	2	72°26′16.19″	3°30′29.27″	186
37	S.103	High plain	Manacacías	13	4.0–5.5	Clay alluvium	>2300	>2	99	1	72°26′16.19″	3°30′29.27″	186
38	S.104	High plain	Manacacías	20	4.0–5.5	Clay alluvium	>2300	>2	99	1	72°26′16.19″	3°30′29.27″	186
39	S.105	High plain	Manacacías	21	4.0–5.5	Clay alluvium	>2300	>2	96	4	72°26′16.19″	3°30′29.27″	186
40	S.107	High plain	Manacacías	17	4.0–5.5	Clay alluvium	>2300	>2	99	1	72°26′16.19″	3°30′29.27″	186
41	S.161	High plain	Manacacías	15	4.0–5.5	Clay alluvium	>2300	>2	98	2	73°2′16.25″	3°52′50.1″	180
42	S.162	High plain	Manacacías	21	4.0–5.5	Clay alluvium	>2300	>2	100	0	73°3′16.25″	2°27′10.54″	180
43	S.163	High plain	Manacacías	13	4.0–5.5	Clay alluvium	>2300	>2	53	47	73°3′16.25″	2°27′10.54″	180
44	S.164	High plain	Manacacías	20	4.0–5.5	Clay alluvium	>2300	>2	98	2	73°2′16.25″	3°52′50.1″	180
45	S.165	High plain	Manacacías	21	4.0–5.5	Clay alluvium	>2300	>2	96	4	73°46′16.25″	2°4′9.7″	180
46	S.167	High plain	Manacacías	17	4.0–5.5	Clay alluvium	>2300	>2	99	1	73°46′16.25″	2°4′9.7″	180
47	S.108	High plain	Manacacías	15	4.0–5.5	Clay alluvium	>2300	>2	100	0	72°26′16.19″	3°30′29.27″	186
48	S.109	High plain	Manacacías	23	4.0–5.5	Clay alluvium	>2300	>2	98	2	72°26′16.19″	3°30′29.27″	186
49	S.110	High plain	Manacacías	16	4.0–5.5	Clay alluvium	>2300	>2	100	0	72°33′16.19″	3°32′19.4″	186
50	S.168	High plain	Manacacías	15	4.0–5.5	Clay alluvium	>2300	>2	100	0	73°46′16.25″	2°4′9.7″	180
51	S.169	High plain	Manacacías	23	4.0–5.5	Clay alluvium	>2300	>2	98	2	73°46′16.25″	2°4′9.7″	180
52	S.170	High plain	Manacacías	16	4.0–5.5	Clay alluvium	>2300	>2	100	0	73°12′16.25″	2°28′6.28″	180
53	S.160	High plain	Manacacías	7	4.0–5.5	Clay alluvium	>2300	>2	99	1	73°2′16.24″	3°52′50.1″	216
54	S.122	High plain	Manacacías	20	4.0–5.5	Clay alluvium	>2300	>2	98	2	72°23′16.21″	3°29′20.47″	169
55	S.124	High plain	Manacacías	15	4.0–5.5	Clay alluvium	>2300	>2	100	0	72°23′16.21″	3°29′20.47″	169
56	S.121	High plain	Manacacías	17	4.0–5.5	Clay alluvium	>2300	>2	100	0	72°23′16.21″	3°29′20.47″	169
57	S.126	High plain	Manacacías	16	4.0–5.5	Clay alluvium	>2300	>2	100	0	72°23′16.21″	3°29′20.47″	169
58	S.128	High plain	Manacacías	16	4.0–5.5	Clay alluvium	>2300	>2	100	0	72°23′16.21″	3°29′20.47″	169
59	S.129	High plain	Manacacías	16	4.0–5.5	Clay alluvium	>2300	>2	98	2	72°23′16.21″	3°29′17.2″	169
60	S.123	High plain	Manacacías	15	4.0–5.5	Clay alluvium	>2300	>2	100	0	72°23′16.21″	3°29′20.47″	169
61	S.152	High plain	Manacacías	14	4.0–5.5	Clay alluvium	>2300	>2	100	0	72°28′16.24″	3°35′24.4″	216
62	S.154	High plain	Manacacías	10	4.0–5.5	Clay alluvium	>2300	>2	99	1	72°21′16.24″	2°38′31.37″	216
63	S.157	High plain	Manacacías	6	4.0–5.5	Clay alluvium	>2300	>2	100	0	72°21′16.24″	2°38′31.37″	216
64	S.158	High plain	Manacacías	8	4.0–5.5	Clay alluvium	>2300	>2	100	0	72°25′16.24″	2°30′55.39″	216
65	S.153	High plain	Manacacías	13	4.0–5.5	Clay alluvium	>2300	>2	100	0	72°21′16.24″	2°38′31.37″	216
66	S.151	High plain	Manacacías	11	4.0–5.5	Clay alluvium	>2300	>2	99	1	72°28′16.24″	3°35′23.7″	216
67	S.155	High plain	Manacacías	12	4.0–5.5	Clay alluvium	>2300	>2	99	1	72°21′16.24″	2°38′31.37″	216
68	S.156	High plain	Manacacías	14	4.0–5.5	Clay alluvium	>2300	>2	100	0	72°21′16.24″	2°38′31.37″	216
69	S.125	High plain	Manacacías	21	4.0–5.5	Clay alluvium	>2300	>2	100	0	72°23′16.21″	3°29′20.47″	169
70	S.127	High plain	Manacacías	19	4.0–5.5	Clay alluvium	>2300	>2	98	2	72°23′16.21″	3°29′20.47″	169
71	S.159	High plain	Manacacías	10	4.0–5.5	Clay alluvium	>2300	>2	100	0	72°25′16.24″	2°30′55.39″	216
72	S.41	High plain	Manacacías	14	4.0–5.5	Clay alluvium	>2300	>2	90	10	72°23′16.13″	3°27′19.08″	164
73	S.42	High plain	Manacacías	11	4.0–5.5	Clay alluvium	>2300	>2	94	6	72°23′16.13″	3°27′19.08″	164
74	S.43	High plain	Manacacías	18	4.0–5.5	Clay alluvium	>2300	>2	91	9	72°23′16.13″	3°27′19.08″	164
75	S.44	High plain	Manacacías	16	4.0–5.5	Clay alluvium	>2300	>2	96	4	72°23′16.13″	3°27′19.08″	164
76	S.45	High plain	Manacacías	12	4.0–5.5	Clay alluvium	>2300	>2	98	2	72°23′16.13″	3°27′19.08″	164
77	S.46	High plain	Manacacías	10	4.0–5.5	Clay alluvium	>2300	>2	86	14	72°23′16.13″	3°27′19.08″	169
78	S.49	High plain	Manacacías	12	4.0–5.5	Clay alluvium	>2300	>2	89	11	72°23′16.13″	3°27′19.08″	169
79	S.51	High plain	Manacacías	7	4.0–5.5	Clay alluvium	>2300	>2	67	33	72°23′16.14″	3°27′18.14″	180
80	S.52	High plain	Manacacías	11	4.0–5.5	Clay alluvium	>2300	>2	72	28	72°23′16.14″	3°27′18.14″	180
81	S.54	High plain	Manacacías	7	4.0–5.5	Clay alluvium	>2300	>2	87	13	72°23′16.14″	3°27′18.14″	180
82	S.55	High plain	Manacacías	9	4.0–5.5	Clay alluvium	>2300	>2	85	15	72°23′16.14″	3°27′18.14″	180
83	S.58	High plain	Manacacías	10	4.0–5.5	Clay alluvium	>2300	>2	90	10	72°23′16.14″	3°27′18.14″	183
84	S.59	High plain	Manacacías	7	4.0–5.5	Clay alluvium	>2300	>2	91	9	72°23′16.14″	3°27′18.14″	183
85	S.31	High plain	Manacacías	11	4.0–5.5	Clay alluvium	>2300	>2	62	38	72°24′16.12″	3°26′45.02″	164
86	S.36	High plain	Manacacías	8	4.0–5.5	Clay alluvium	>2300	>2	81	19	72°24′16.12″	3°26′45.02″	169
87	S.38	High plain	Manacacías	7	4.0–5.5	Clay alluvium	>2300	>2	95	5	72°24′16.12″	3°26′45.02″	169
88	S.40	High plain	Manacacías	7	4.0–5.5	Clay alluvium	>2300	>2	91	9	72°24′16.12″	3°26′45.02″	169
89	S.86	High plain	Manacacías	15	4.0–5.5	Clay alluvium	>2300	>2	97	3	72°23′16.17″	3°29′20.47″	173
90	S.111	High plain	Manacacías	17	4.0–5.5	Clay alluvium	>2300	>2	92	8	72°36′16.2″	3°32′18.7″	186
91	S.113	High plain	Manacacías	17	4.0–5.5	Clay alluvium	>2300	>2	97	3	72°36′16.2″	3°34′28.93″	186
92	S.116	High plain	Manacacías	9	4.0–5.5	Clay alluvium	>2300	>2	95	5	72°28′16.2″	3°37′34.8″	186
93	S.117	High plain	Manacacías	8	4.0–5.5	Clay alluvium	>2300	>2	85	15	72°28′16.2″	3°38′13.3″	186
94	S.143	High plain	Manacacías	14	4.0–5.5	Clay alluvium	>2300	>2	92	8	72°28′16.23″	3°35′40.6″	197
95	S.118	High plain	Manacacías	13	4.0–5.5	Clay alluvium	>2300	>2	94	6	72°29′16.2″	3°38′24.5″	186
96	S.119	High plain	Manacacías	14	4.0–5.5	Clay alluvium	>2300	>2	96	4	72°23′16.2″	3°29′20.47″	186
97	S.120	High plain	Manacacías	25	4.0–5.5	Clay alluvium	>2300	>2	81	19	72°23′16.2″	3°29′20.47″	186
98	S.149	High plain	Manacacías	12	4.0–5.5	Clay alluvium	>2300	>2	88	12	72°35′16.23″	3°35′45.6″	197
99	S.130	High plain	Manacacías	14	4.0–5.5	Clay alluvium	>2300	>2	98	2	72°23′16.21″	3°29′17.2″	169
100	S.50	High plain	Manacacías	12	4.0–5.5	Clay alluvium	>2300	>2	92	8	72°23′16.13″	3°27′19.08″	169
101	S.57	High plain	Manacacías	9	4.0–5.5	Clay alluvium	>2300	>2	95	5	72°23′16.14″	3°27′18.14″	183
102	S.79	High plain	Manacacías	10	4.0–5.5	Clay alluvium	>2300	>2	84	16	72°26′16.16″	3°30′29.27″	199
103	S.70	High plain	Manacacías	8	4.0–5.5	Clay alluvium	>2300	>2	88	12	72°26′16.15″	3°30′35.75″	200
104	S.72	High plain	Manacacías	12	4.0–5.5	Clay alluvium	>2300	>2	95	5	72°26′16.16″	3°30′29.27″	200
105	S.74	High plain	Manacacías	11	4.0–5.5	Clay alluvium	>2300	>2	94	6	72°26′16.16″	3°30′29.27″	200
106	S.76	High plain	Manacacías	14	4.0–5.5	Clay alluvium	>2300	>2	95	5	72°26′16.16″	3°30′29.27″	199
107	S.80	High plain	Manacacías	8	4.0–5.5	Clay alluvium	>2300	>2	90	10	72°26′16.16″	3°30′29.27″	167
108	S.138	High plain	Manacacías	12	4.0–5.5	Clay alluvium	>2300	>2	97	3	72°23′16.22″	3°29′17.2″	177
109	S.139	High plain	Manacacías	11	4.0–5.5	Clay alluvium	>2300	>2	80	20	72°36′16.22″	3°34′18.4″	177
110	S.136	High plain	Manacacías	16	4.0–5.5	Clay alluvium	>2300	>2	96	4	72°23′16.22″	3°29′17.2″	177
111	S.132	High plain	Manacacías	17	4.0–5.5	Clay alluvium	>2300	>2	93	7	72°23′16.22″	3°29′17.2″	177
112	S.133	High plain	Manacacías	13	4.0–5.5	Clay alluvium	>2300	>2	99	1	72°23′16.22″	3°29′17.2″	177
113	S.134	High plain	Manacacías	14	4.0–5.5	Clay alluvium	>2300	>2	80	20	72°23′16.22″	3°29′17.2″	177
114	S.135	High plain	Manacacías	17	4.0–5.5	Clay alluvium	>2300	>2	97	3	72°23′16.22″	3°29′17.2″	177
115	S.137	High plain	Manacacías	17	4.0–5.5	Clay alluvium	>2300	>2	94	6	72°23′16.22″	3°29′17.2″	177
116	S.77	High plain	Manacacías	12	4.0–5.5	Clay alluvium	>2300	>2	97	3	72°26′16.16″	3°30′29.27″	199
117	S.141	High plain	Manacacías	15	4.0–5.5	Clay alluvium	>2300	>2	97	3	72°36′16.23″	3°34′20.7″	197
118	S.142	High plain	Manacacías	11	4.0–5.5	Clay alluvium	>2300	>2	92	8	72°28′16.23″	3°33′42.5″	197
119	S.144	High plain	Manacacías	13	4.0–5.5	Clay alluvium	>2300	>2	92	8	72°28′16.23″	3°35′38.9″	197
120	S.20	High plain	Manacacías	8	4.0–5.5	Clay alluvium	>2300	>2	96	4	72°27′16.1″	3°29′52.3″	172
121	S.85	High plain	Manacacías	9	4.0–5.5	Clay alluvium	>2300	>2	90	10	72°23′16.17″	3°29′20.47″	173
122	S.82	High plain	Manacacías	13	4.0–5.5	Clay alluvium	>2300	>2	94	6	72°23′16.17″	3°29′20.47″	167
123	S.87	High plain	Manacacías	13	4.0–5.5	Clay alluvium	>2300	>2	97	3	72°23′16.17″	3°29′20.47″	173
124	S.89	High plain	Manacacías	11	4.0–5.5	Clay alluvium	>2300	>2	91	9	72°23′16.17″	3°29′20.47″	173
125	S.98	High plain	Manacacías	6	4.0–5.5	Clay alluvium	>2300	>2	87	13	72°23′16.18″	3°29′17.2″	170
126	S.16	High plain	Manacacías	6	4.0–5.5	Clay alluvium	>2300	>2	80	20	72°27′16.1″	3°29′52.3″	172
127	S.32	High plain	Manacacías	9	4.0–5.5	Clay alluvium	>2300	>2	96	4	72°24′16.12″	3°26′45.02″	164
128	S.35	High plain	Manacacías	5	4.0–5.5	Clay alluvium	>2300	>2	92	8	72°24′16.12″	3°26′45.02″	164
129	S.37	High plain	Manacacías	5	4.0–5.5	Clay alluvium	>2300	>2	96	4	72°24′16.12″	3°26′45.02″	169
130	S.81	High plain	Manacacías	10	4.0–5.5	Clay alluvium	>2300	>2	87	13	72°23′16.17″	3°29′20.47″	167
131	S.90	High plain	Manacacías	8	4.0–5.5	Clay alluvium	>2300	>2	95	5	72°23′16.17″	3°29′20.47″	174
132	S.145	High plain	Manacacías	14	4.0–5.5	Clay alluvium	>2300	>2	93	7	72°28′16.23″	3°35′34.6″	197
133	S.146	High plain	Manacacías	13	4.0–5.5	Clay alluvium	>2300	>2	97	3	72°28′16.23″	3°35′37″	197
134	S.147	High plain	Manacacías	14	4.0–5.5	Clay alluvium	>2300	>2	99	1	72°33′16.23″	3°31′44″	197
135	S.148	High plain	Manacacías	15	4.0–5.5	Clay alluvium	>2300	>2	96	4	72°35′16.23″	3°35′46.3″	197
136	S.150	High plain	Manacacías	10	4.0–5.5	Clay alluvium	>2300	>2	85	15	72°35′16.23″	3°35′46.3″	197
137	S.178	High plain	Manacacías	5	4.0–5.5	Clay alluvium	>2300	>2	96	4	72°33′16.26″	3°32′19.4″	234
138	S.183	High plain	Manacacías	10	4.0–5.5	Clay alluvium	>2300	>2	82	18	72°36′16.27″	3°32′18.7″	230
139	S.184	High plain	Manacacías	10	4.0–5.5	Clay alluvium	>2300	>2	92	8	72°28′16.27″	3°37′34.8″	201
140	S.185	High plain	Manacacías	10	4.0–5.5	Clay alluvium	>2300	>2	88	12	72°28′16.27″	3°38′13.3″	208
141	S.188	High plain	Manacacías	11	4.0–5.5	Clay alluvium	>2300	>2	85	15	72°36′16.27″	3°32′18.6″	222
142	S.33	High plain	Manacacías	8	4.0–5.5	Clay alluvium	>2300	>2	96	4	72°24′16.12″	3°26′45.02″	164
143	S.93	High plain	Manacacías	7	4.0–5.5	Clay alluvium	>2300	>2	85	15	72°23′16.18″	3°29′17.2″	174
144	S.114	High plain	Manacacías	11	4.0–5.5	Clay alluvium	>2300	>2	91	9	72°36′16.2″	3°34′30.47″	186
145	S.63	High plain	Manacacías	8	4.0–5.5	Clay alluvium	>2300	>2	97	3	72°26′16.15″	3°30′35.75″	183
146	S.68	High plain	Manacacías	7	4.0–5.5	Clay alluvium	>2300	>2	84	16	72°26′16.15″	3°30′35.75″	180
147	S.69	High plain	Manacacías	6	4.0–5.5	Clay alluvium	>2300	>2	85	15	72°26′16.15″	3°30′35.75″	180
148	S.181	High plain	Manacacías	8	4.0–5.5	Clay alluvium	>2300	>2	89	11	72°36′16.27″	3°34′16.1″	225
149	S.191	High plain	Manacacías	3	4.0–5.5	Clay alluvium	>2300	>2	82	18	72°32′16.28″	3°41′11.4″	200
150	S.83	High plain	Manacacías	6	4.0–5.5	Clay alluvium	>2300	>2	87	13	72°23′16.17″	3°29′20.47″	167
151	S.84	High plain	Manacacías	10	4.0–5.5	Clay alluvium	>2300	>2	93	7	72°23′16.17″	3°29′20.47″	167
152	S.34	High plain	Manacacías	7	4.0–5.5	Clay alluvium	>2300	>2	74	26	72°24′16.12″	3°26′45.02″	164
153	S.88	High plain	Manacacías	8	4.0–5.5	Clay alluvium	>2300	>2	90	10	72°23′16.17″	3°29′20.47″	173
154	S.131	High plain	Manacacías	14	4.0–5.5	Clay alluvium	>2300	>2	86	14	72°23′16.22″	3°29′17.2″	177
155	S.171	High plain	Manacacías	5	4.0–5.5	Clay alluvium	>2300	>2	97	3	72°35′16.26″	3°35′48.88″	177
156	S.189	High plain	Manacacías	6	4.0–5.5	Clay alluvium	>2300	>2	71	29	72°34′16.27″	3°41′59.3″	230
157	S.175	High plain	Manacacías	3	4.0–5.5	Clay alluvium	>2300	>2	80	20	72°36′16.26″	3°34′30.47″	177
158	S.182	High plain	Manacacías	8	4.0–5.5	Clay alluvium	>2300	>2	88	12	72°36′16.27″	3°34′20.7″	235
159	S.172	High plain	Manacacías	7	4.0–5.5	Clay alluvium	>2300	>2	73	27	72°35′16.26″	3°50′51.4″	176
160	S.179	High plain	Manacacías	8	4.0–5.5	Clay alluvium	>2300	>2	89	11	72°36′16.26″	3°34′17.5″	225
161	S.177	High plain	Manacacías	15	4.0–5.5	Clay alluvium	>2300	>2	70	30	72°36′16.26″	3°34′18.4″	233
162	S.180	High plain	Manacacías	6	4.0–5.5	Clay alluvium	>2300	>2	69	31	72°36′16.26″	3°34′16.8″	224
163	S.140	High plain	Manacacías	9	4.0–5.5	Clay alluvium	>2300	>2	87	13	72°36′16.22″	3°34′16.1″	177
164	S.Ar.21	Alluvial plain	Arauca	14	3.6–5.5	Clay alluvium	<2000	<2	100	0	69°54′28.8″	6°12′40.03″	107
165	S.Ar.22	Alluvial plain	Arauca	16	3.6–5.5	Clay alluvium	<2000	<2	82	18	69°54′28.47″	6°12′39.99″	109
166	S.Ar.27	Alluvial plain	Arauca	12	3.6–5.5	Clay alluvium	<2000	<2	75	25	69°54′27.28″	6°12′37.47″	114
167	S.Ar.28	Alluvial plain	Arauca	15	3.6–5.5	Clay alluvium	<2000	<2	73	27	69°54′28.07″	6°12′36.97″	116
168	S.Ar.31	Alluvial plain	Arauca	11	3.6–5.5	Clay alluvium	<2000	<2	83	17	69°53′21.87″	6°13′3.28″	107
169	S.Ar.24	Alluvial plain	Arauca	18	3.6–5.5	Clay alluvium	<2000	<2	90	10	69°54′29.08″	6°12′39.16″	113
170	S.Ar.70	Alluvial plain	Arauca	16	3.6–5.5	Clay alluvium	<2000	<2	98	2	70°25′38.17″	6°23′37.93″	106
171	S.Ar.29	Alluvial plain	Arauca	12	3.6–5.5	Clay alluvium	<2000	<2	76	24	69°54′27.57″	6°12′36.79″	117
172	S.Ar.23	Alluvial plain	Arauca	21	3.6–5.5	Clay alluvium	<2000	<2	98	2	69°54′28.18″	6°12′39.6″	111
173	S.Ar.25	Alluvial plain	Arauca	11	3.6–5.5	Clay alluvium	<2000	<2	75	25	69°54′28.58″	6°12′38.7″	110
174	S.Ar.32	Alluvial plain	Arauca	18	3.6–5.5	Clay alluvium	<2000	<2	100	0	69°53′21.19″	6°13′3.79″	107
175	S.Ar.33	Alluvial plain	Arauca	10	3.6–5.5	Clay alluvium	<2000	<2	71	29	69°53′20.97″	6°13′4.08″	107
176	S.Ar.34	Alluvial plain	Arauca	14	3.6–5.5	Clay alluvium	<2000	<2	86	14	69°53′20.86″	6°13′4.69″	107
177	S.Ar.36	Alluvial plain	Arauca	12	3.6–5.5	Clay alluvium	<2000	<2	94	6	69°53′21.3″	6°13′5.7″	108
178	S.Ar.38	Alluvial plain	Arauca	12	3.6–5.5	Clay alluvium	<2000	<2	87	13	69°53′20.79″	6°13′5.98″	108
179	S.Ar.39	Alluvial plain	Arauca	13	3.6–5.5	Clay alluvium	<2000	<2	94	6	69°53′20.18″	6°13′6.27″	108
180	S.Ar.4	Alluvial plain	Arauca	9	3.6–5.5	Clay alluvium	<2000	<2	52	48	69°54′56.7″	6°11′39.69″	103
181	S.Ar.40	Alluvial plain	Arauca	16	3.6–5.5	Clay alluvium	<2000	<2	93	7	69°53′20.39″	6°13′6.59″	108
182	S.Ar.35	Alluvial plain	Arauca	10	3.6–5.5	Clay alluvium	<2000	<2	77	23	69°53′20.68″	6°13′5.19″	107
183	S.Ar.37	Alluvial plain	Arauca	9	3.6–5.5	Clay alluvium	<2000	<2	81	19	69°53′21.48″	6°13′5.88″	108
184	S.Ar.51	Alluvial plain	Arauca	6	3.6–5.5	Clay alluvium	<2000	<2	49	51	69°52′35.18″	6°13′21.46″	100
185	S.Ar.83	Alluvial plain	Arauca	14	3.6–5.5	Clay alluvium	<2000	<2	94	6	70°51′20.01″	6°23′5.31″	126
186	S.Ar.54	Alluvial plain	Arauca	9	3.6–5.5	Clay alluvium	<2000	<2	69	31	69°52′25.89″	6°13′22.79″	100
187	S.Ar.55	Alluvial plain	Arauca	12	3.6–5.5	Clay alluvium	<2000	<2	67	33	69°52′23.19″	6°13′21.97″	98
188	S.Ar.56	Alluvial plain	Arauca	11	3.6–5.5	Clay alluvium	<2000	<2	58	42	69°52′21.97″	6°13′23.48″	98
189	S.Ar.57	Alluvial plain	Arauca	9	3.6–5.5	Clay alluvium	<2000	<2	59	41	69°52′20.38″	6°13′23.7″	98
190	S.Ar.59	Alluvial plain	Arauca	11	3.6–5.5	Clay alluvium	<2000	<2	85	15	69°51′58.49″	6°13′23.88″	99
191	S.Ar.6	Alluvial plain	Arauca	7	3.6–5.5	Clay alluvium	<2000	<2	23	77	69°54′55.97″	6°11′40.27″	104
192	S.Ar.10	Alluvial plain	Arauca	9	3.6–5.5	Clay alluvium	<2000	<2	57	43	69°54′54.39″	6°11′41.67″	106
193	S.Ar.18	Alluvial plain	Arauca	8	3.6–5.5	Clay alluvium	<2000	<2	100	0	69°53′15.28″	6°12′15.76″	108
194	S.Ar.3	Alluvial plain	Arauca	11	3.6–5.5	Clay alluvium	<2000	<2	35	65	69°54′56.98″	6°11′39.3″	103
195	S.Ar.71	Alluvial plain	Arauca	28	3.6–5.5	Clay alluvium	<2000	<2	94	6	70°45′7.01″	6°24′20.33″	155
196	S.Ar.72	Alluvial plain	Arauca	33	3.6–5.5	Clay alluvium	<2000	<2	97	3	70°45′7.92″	6°24′20.44″	115
197	S.Ar.8	Alluvial plain	Arauca	14	3.6–5.5	Clay alluvium	<2000	<2	51	49	69°54′55.29″	6°11′40.99″	105
198	S.Ar.14	Alluvial plain	Arauca	8	3.6–5.5	Clay alluvium	<2000	<2	49	51	69°53′13.99″	6°12′15.47″	106
199	S.Ar.17	Alluvial plain	Arauca	10	3.6–5.5	Clay alluvium	<2000	<2	63	37	69°53′15″	6°12′15.87″	106
200	S.Ar.2	Alluvial plain	Arauca	10	3.6–5.5	Clay alluvium	<2000	<2	71	29	69°54′57.27″	6°11′38.97″	102
201	S.Ar.5	Alluvial plain	Arauca	11	3.6–5.5	Clay alluvium	<2000	<2	75	25	69°54′56.26″	6°11′39.98″	104
202	S.Ar.13	Alluvial plain	Arauca	11	3.6–5.5	Clay alluvium	<2000	<2	49	51	69°53′13.48″	6°12′15.58″	108
203	S.Ar.7	Alluvial plain	Arauca	10	3.6–5.5	Clay alluvium	<2000	<2	52	48	69°54′55.69″	6°11′40.66″	105
204	S.Ar.9	Alluvial plain	Arauca	9	3.6–5.5	Clay alluvium	<2000	<2	95	5	69°54′54.97″	6°11′41.27″	106
205	S.Ar.47	Alluvial plain	Arauca	14	3.6–5.5	Clay alluvium	<2000	<2	89	11	69°53′4.09″	6°13′17.18″	102
206	S.Ar.73	Alluvial plain	Arauca	16	3.6–5.5	Clay alluvium	<2000	<2	90	10	70°48′39.24″	6°23′49.7″	119
207	S.Ar.80	Alluvial plain	Arauca	18	3.6–5.5	Clay alluvium	<2000	<2	91	9	70°49′57.57″	6°21′43.16″	126
208	S.Ar.84	Alluvial plain	Arauca	16	3.6–5.5	Clay alluvium	<2000	<2	89	11	70°42′46.51″	6°25′48.54″	119
209	S.Ar.87	Alluvial plain	Arauca	19	3.6–5.5	Clay alluvium	<2000	<2	71	29	70°43′1.52″	6°26′18.09″	115
210	S.Ar.89	Alluvial plain	Arauca	11	3.6–5.5	Clay alluvium	<2000	<2	93	7	70°43′11.42″	6°26′11.07″	118
211	S.Ar.90	Alluvial plain	Arauca	19	3.6–5.5	Clay alluvium	<2000	<2	79	21	70°50′37.93″	6°22′59.7″	123
212	S.Ar.92	Alluvial plain	Arauca	15	3.6–5.5	Clay alluvium	<2000	<2	79	21	70°49′29.92″	6°23′0.13″	125
213	S.Ar.94	Alluvial plain	Arauca	6	3.6–5.5	Clay alluvium	<2000	<2	75	25	70°49′15.34″	6°23′3.91″	127
214	S.Ar.95	Alluvial plain	Arauca	19	3.6–5.5	Clay alluvium	<2000	<2	69	31	70°49′2.85″	6°23′4.81″	124
215	S.Ar.96	Alluvial plain	Arauca	14	3.6–5.5	Clay alluvium	<2000	<2	69	31	70°49′35.14″	6°22′59.41″	121
216	S.Ar. 49	Alluvial plain	Arauca	16	3.6–5.5	Clay alluvium	<2000	<2	97	3	69°53′0.67″	6°13′19.66″	101
217	S.Ar. 50	Alluvial plain	Arauca	14	3.6–5.5	Clay alluvium	<2000	<2	75	25	69°52′59.7″	6°13′20.27″	101
218	S.Ar. 16	Alluvial plain	Arauca	8	3.6–5.5	Clay alluvium	<2000	<2	64	36	69°53′14.67″	6°12′15.47″	106
219	S.Ar. 11	Alluvial plain	Arauca	9	3.6–5.5	Clay alluvium	<2000	<2	67	33	69°53′12.19″	6°12′15.76″	109
220	S.Ar. 1	Alluvial plain	Arauca	10	3.6–5.5	Clay alluvium	<2000	<2	92	8	69°54′57.49″	6°11′38.79″	102
221	S.Ar. 12	Alluvial plain	Arauca	9	3.6–5.5	Clay alluvium	<2000	<2	86	14	69°53′12.98″	6°12′15.87″	109
222	S.Ar. 15	Alluvial plain	Arauca	9	3.6–5.5	Clay alluvium	<2000	<2	50	50	69°53′14.49″	6°12′15.37″	108
223	S.Ar. 19	Alluvial plain	Arauca	7	3.6–5.5	Clay alluvium	<2000	<2	48	52	69°53′15.89″	6°12′15.58″	105
224	S.Ar. 20	Alluvial plain	Arauca	15	3.6–5.5	Clay alluvium	<2000	<2	61	39	69°53′16.36″	6°12′15.69″	107
225	S.Ar. 42	Alluvial plain	Arauca	7	3.6–5.5	Clay alluvium	<2000	<2	45	55	69°53′9.09″	6°13′12.17″	107
226	S.Ar. 41	Alluvial plain	Arauca	12	3.6–5.5	Clay alluvium	<2000	<2	90	10	69°53′10.06″	6°13′9.19″	107
227	S.Ar. 43	Alluvial plain	Arauca	14	3.6–5.5	Clay alluvium	<2000	<2	95	5	69°53′7.29″	6°13′15.67″	106
228	S.Ar. 44	Alluvial plain	Arauca	13	3.6–5.5	Clay alluvium	<2000	<2	100	0	69°53′6.89″	6°13′15.38″	106
229	S.Ar. 45	Alluvial plain	Arauca	15	3.6–5.5	Clay alluvium	<2000	<2	100	0	69°53′5.67″	6°13′17.86″	102
230	S.Ar. 48	Alluvial plain	Arauca	17	3.6–5.5	Clay alluvium	<2000	<2	100	0	69°53′1.89″	6°13′19.09″	101
231	S.Ar. 68	Alluvial plain	Arauca	38	3.6–5.5	Clay alluvium	<2000	<2	100	0	70°36′44.1″	6°59′3.69″	107
232	S.Ar. 85	Alluvial plain	Arauca	17	3.6–5.5	Clay alluvium	<2000	<2	90	10	70°42′44.82″	6°25′58.83″	118
233	S.Ar. 46	Alluvial plain	Arauca	13	3.6–5.5	Clay alluvium	<2000	<2	72	28	69°53′4.38″	6°13′18.58″	102
234	S.Ar. 52	Alluvial plain	Arauca	10	3.6–5.5	Clay alluvium	<2000	<2	62	38	69°52′27.37″	6°13′22.08″	100
235	S.Ar. 53	Alluvial plain	Arauca	12	3.6–5.5	Clay alluvium	<2000	<2	73	27	69°52′26.29″	6°13′21.89″	100
236	S.Ar. 58	Alluvial plain	Arauca	12	3.6–5.5	Clay alluvium	<2000	<2	84	16	69°52′19.99″	6°13′23.98″	98
237	S.Ar. 60	Alluvial plain	Arauca	14	3.6–5.5	Clay alluvium	<2000	<2	57	43	69°51′56.19″	6°13′24.27″	97
238	S.Ar. 77	Alluvial plain	Arauca	14	3.6–5.5	Clay alluvium	<2000	<2	98	2	70°49′15.99″	6°20′7.29″	127
239	S.Ar. 79	Alluvial plain	Arauca	13	3.6–5.5	Clay alluvium	<2000	<2	100	0	70°50′32.06″	6°23′3.33″	124
240	S.Ar. 78	Alluvial plain	Arauca	16	3.6–5.5	Clay alluvium	<2000	<2	96	4	70°50′30.98″	6°23′0.92″	124
241	S.Ar. 69	Alluvial plain	Arauca	9	3.6–5.5	Clay alluvium	<2000	<2	82	18	70°25′16.42″	6°23′49.95″	106
242	S.Ar. 76	Alluvial plain	Arauca	10	3.6–5.5	Clay alluvium	<2000	<2	97	3	70°50′22.77″	6°22′31.65″	132
243	S.Ar. 81	Alluvial plain	Arauca	18	3.6–5.5	Clay alluvium	<2000	<2	100	0	70°50′30.91″	6°22′3.57″	123
244	S.Ar. 88	Alluvial plain	Arauca	13	3.6–5.5	Clay alluvium	<2000	<2	100	0	70°43′6.24″	6°26′20.43″	115
245	S.Ar. 93	Alluvial plain	Arauca	11	3.6–5.5	Clay alluvium	<2000	<2	98	2	70°49′23.91″	6°23′0.92″	124
246	S.Ar. 62	Alluvial plain	Arauca	17	3.6–5.5	Clay alluvium	<2000	<2	94	6	70°41′37.78″	7°3′34.59″	126
247	S.Ar. 66	Alluvial plain	Arauca	7	3.6–5.5	Clay alluvium	<2000	<2	96	4	70°43′5.91″	7°1′31″	101
248	S.Ar. 65	Alluvial plain	Arauca	6	3.6–5.5	Clay alluvium	<2000	<2	99	1	70°41′49.77″	7°3′42.08″	102
249	S.Ar. 74	Alluvial plain	Arauca	19	3.6–5.5	Clay alluvium	<2000	<2	63	37	70°49′39.39″	6°23′32.2″	116
250	S.Ar. 61	Alluvial plain	Arauca	17	3.6–5.5	Clay alluvium	<2000	<2	70	30	70°38′46.89″	7°3′57.67″	123
251	S.Ar. 64	Alluvial plain	Arauca	18	3.6–5.5	Clay alluvium	<2000	<2	100	0	70°34′56.06″	7°4′26.18″	103
252	S.Ar. 63	Alluvial plain	Arauca	14	3.6–5.5	Clay alluvium	<2000	<2	98	2	70°25′37.66″	6°21′32.18″	100
253	S.Ar. 82	Alluvial plain	Arauca	14	3.6–5.5	Clay alluvium	<2000	<2	99	1	70°50′6.07″	6°22′23.73″	122
254	S.Ar. 86	Alluvial plain	Arauca	11	3.6–5.5	Clay alluvium	<2000	<2	29	71	70°42′40.89″	6°25′22.4″	118
255	S.Ar. 91	Alluvial plain	Arauca	13	3.6–5.5	Clay alluvium	<2000	<2	99	1	70°50′36.67″	6°21′43.73″	124
256	CAS.33	Alluvial plain	Casanare	4	4.3–6.5	Sandy alluvium	2000–2100	<2	80	20	71°25′38.9″	5°32′29.3″	149
257	CAS.58	Alluvial plain	Casanare	8	4.3–6.5	Sandy alluvium	2000–2100	<2	90	10	71°25′35.6″	5°32′30.1″	148
258	CAS.35	Alluvial plain	Casanare	4	4.3–6.5	Sandy alluvium	2000–2100	<2	75	25	71°26′24.7″	5°31′46.0″	157
259	CAS.36	Alluvial plain	Casanare	4	4.3–6.5	Sandy alluvium	2000–2100	<2	75	25	71°26′23.6″	5°31′49.4″	154
260	CAS.34	Alluvial plain	Casanare	4	4.3–6.5	Sandy alluvium	2000–2100	<2	75	25	71°26′26.4″	5°31′44.9″	153
261	CAS.45	Alluvial plain	Casanare	16	4.3–6.5	Sandy alluvium	2000–2100	<2	97	3	71°26′03.2″	5°31′34.2″	149
262	CAS.59	Alluvial plain	Casanare	16	4.3–6.5	Sandy alluvium	2000–2100	<2	100	0	71°25′36.8″	5°32′29.9″	151
263	CAS.38	Alluvial plain	Casanare	6	4.3–6.5	Sandy alluvium	2000–2100	<2	70	30	71°42′10.1″	5°25′51.9″	165
264	CAS.46	Alluvial plain	Casanare	6	4.3–6.5	Sandy alluvium	2000–2100	<2	100	0	71°42′05.1″	5°25′49.8″	164
265	CAS.55	Alluvial plain	Casanare	12	4.3–6.5	Sandy alluvium	2000–2100	<2	100	0	71°3′48.521″	5°21′34.307″	138
266	CAS.57	Alluvial plain	Casanare	8	4.3–6.5	Sandy alluvium	2000–2100	<2	95	5	71°3′44.396″	5°21′33.200″	134
267	CAS.54	Alluvial plain	Casanare	9	4.3–6.5	Sandy alluvium	2000–2100	<2	100	0	71°1′45.745″	5°21′42.960″	118
268	CAS.48	Alluvial plain	Casanare	26	4.3–6.5	Sandy alluvium	2000–2100	<2	99	1	71°42′15.8″	5°26′51.2″	174
269	CAS.49	Alluvial plain	Casanare	15	4.3–6.5	Sandy alluvium	2000–2100	<2	100	0	71°42′17.1″	5°26′52.6″	172
270	CAS.39	Alluvial plain	Casanare	15	4.3–6.5	Sandy alluvium	2000–2100	<2	95	5	71°42′06.4″	5°25′47.7″	165
271	CAS.50	Alluvial plain	Casanare	13	4.3–6.5	Sandy alluvium	2000–2100	<2	100	0	71°42′27.3″	5°26′45.4″	171
272	CAS.42	Alluvial plain	Casanare	5	4.3–6.5	Sandy alluvium	2000–2100	<2	95	5	71°25′54.8″	5°31′32.3″	155
273	CAS.41	Alluvial plain	Casanare	15	4.3–6.5	Sandy alluvium	2000–2100	<2	100	0	71°26′14.9″	5°31′34.5	154
274	CAS.44	Alluvial plain	Casanare	6	4.3–6.5	Sandy alluvium	2000–2100	<2	85	15	71°26′01.9″	5°31′33.3″	146
275	CAS.40	Alluvial plain	Casanare	7	4.3–6.5	Sandy alluvium	2000–2100	<2	100	0	72°05′25.4″	5°50′34.5″	495
276	CAS.47	Alluvial plain	Casanare	6	4.3–6.5	Sandy alluvium	2000–2100	<2	95	5	70°05′25.1″	5°50′33.2″	500
277	CAS.52	Alluvial plain	Casanare	16	4.3–6.5	Sandy alluvium	2000–2100	<2	100	0	71°37′49.9″	5°23′45.2″	165
278	CAS.51	Alluvial plain	Casanare	8	4.3–6.5	Sandy alluvium	2000–2100	<2	100	0	71°37′52.2″	5°23′44.7″	168
279	CAS.37	Alluvial plain	Casanare	4	4.3–6.5	Sandy alluvium	2000–2100	<2	60	40	71°42′03.6″	5°25′50.8″	165
280	CAS.32	Alluvial plain	Casanare	9	4.3–6.5	Sandy alluvium	2000–2100	<2	100	0	71°24′34.837″	5°27′2.614″	145
281	CAS.53	Alluvial plain	Casanare	13	4.3–6.5	Sandy alluvium	2000–2100	<2	100	0	71°24′35.030″	5°26′59.294″	177
282	CAS.43	Alluvial plain	Casanare	14	4.3–6.5	Sandy alluvium	2000–2100	<2	100	0	71°3′14.061″	5°20′58.366″	121
283	CAS.56	Alluvial plain	Casanare	5	4.3–6.5	Sandy alluvium	2000–2100	<2	95	5	71°3′46.735″	5°21′33.396″	132
284	Met_10	High plain	río Meta	9	4.0–5.5	Clay alluvium	>2300	>2	98	2	67°30′18.89″	06°13′01.01″	82
285	Met_6	High plain	río Meta	16	4.0–5.5	Clay alluvium	>2300	>2	97	3	69°50′26.6″	06°00′26.2″	95
286	Met_9	High plain	río Meta	8	4.0–5.5	Clay alluvium	>2300	>2	85	15	71°19′32.7″	04°47′53.7″	140
287	Met_14	High plain	río Meta	8	4.0–5.5	Clay alluvium	>2300	>2	93	7	67°30′18.25″	06°13′02.10″	80
288	Met_1	High plain	río Meta	11	4.0–5.5	Clay alluvium	>2300	>2	98	2	68°06′33.3″	06°13′07.1″	65
289	Met_29	High plain	río Meta	5	4.0–5.5	Clay alluvium	>2300	>2	97	3	68°06′32.9″	06°13′07.7″	65
290	Met_5	High plain	río Meta	14	4.0–5.5	Clay alluvium	>2300	>2	98	2	68°06′31.2″	06°13′06.7″	70
291	Met_3	High plain	río Meta	14	4.0–5.5	Clay alluvium	>2300	>2	98	2	68°32′14.9″	06°08′27.2″	102
292	Met_4	High plain	río Meta	10	4.0–5.5	Clay alluvium	>2300	>2	93	7	68°49′40.6″	06°09′51.8″	74
